# Subjective Sensations related to Food as Determinants of Snack Choice

**DOI:** 10.3390/foods9030336

**Published:** 2020-03-12

**Authors:** Mette Duerlund, Barbara Vad Andersen, Niki Alexi, Mei Peng, Derek Victor Byrne

**Affiliations:** 1 Department of Food Science, Faculty of Science and Technology, Aarhus University, Agro Food Park 48, DK 8200 Aarhus N, Denmark; barbarav.andersen@food.au.dk (B.V.A.); niki.alexi@food.au.dk (N.A.); derekv.byrne@food.au.dk (D.V.B.); 2 Sensory Neuroscience Laboratory, Department of Food Science, University of Otago, Dunedin 9054, New Zealand; mei.peng@otago.ac.nz

**Keywords:** appetite, satiety, desire, energy, snack, consumer, sensation, wellbeing, choice

## Abstract

Comprehending the complexity and determinants of food choices can help understand facets of the growing obesity epidemic. Focusing on consumers’ subjective sensations as determinants of food choices can provide essential insights into eating behaviors. We explored subjective sensations linked to appetite, desire, wellbeing and energy. This study aims to 1) quantify subjective temporal sensations, and 2) study the effects of these sensations on snack choice. Two-hundred and fifty-three participants (mean age 20.5) evaluated subjective sensations using a visual analogue scale. The choice of one of six snacks was offered to the participants; choices were recorded as implicit choice measures. The results demonstrated that especially sensory specific desire sensations (Salty, Fatty, Sweet desire) affected, either positively or negatively, snack choice. Furthermore, wellbeing sensations (Overall, Mental, Physical wellbeing) also showed significant effects for snack choice. Health-conscious females chose healthy snacks, and males chose unhealthy snacks. Importantly, this research indicates the relevance of subjective sensations in consumer studies that focus on diverse determinants of food choice. Sensory specific desires and wellbeing sensations were notably shown to be important determinants of snack choice. The contribution of different sensations to food choice is imperative, and helps us to understand aspects of snacking behavior. This could have broader implications concerning public health issues and obesity.

## 1. Introduction

We live in a multifaceted world, and the processes around food choices and eating behavior are complex and multifactorial [1,2,3,4]. Seeking to comprehend the complexity and determinants of food choices might help us understand and elucidate some of the underlying facets of the growing obesity epidemic [5,6,7]. Several approaches have been applied to model food choices [2,3,8]. One of the most widely used is the Theory of Planned Behavior (TBP), linking one’s attitudes and behavioral intentions with one’s behavior. TBP functions as a cognitive model and theorizes that we make rational and conscious decisions based on attitudes and intentions [1,3,5]. Yet, stronger attitudes are thought to be more predictive of food choice than weaker, ambivalent attitudes [3,8]. Contrariwise, it has been established that intuitive and subconscious decision-making also play important roles in food-related behavior [4,9]. Often, researchers find inconsistencies or weak associations between intention and actual eating behavior [5,10,11]. For instance, Weijzen et al. (2009) found that 24% of consumers with a healthy snack choice intention actually chose an unhealthy snack instead, showing a discrepancy between intent and action [5]. Psychologist and Nobel Prize winner Daniel Kahneman differentiates between two cognitive systems within decision-making. His groundbreaking two-system view is characterized into a System 1 and a System 2. System 1 is characterized as intuitive, fast and automatic thinking, whereas System 2 is characterized as reasoned, conscious and controlled thinking. It can be said that in basic behavior like eating and drinking, intuitive thinking is a rule rather than an exception [4,9,12]. Actual snack choices are often made impulsively, and maybe even instinctively or unconsciously [13,14]. The realization that much decision making happens unintentionally leads us to reconsider choice measures. More emphasis should be placed on research that shows real behavioral effects, for instance observational studies of actual choices [4]. Measuring actual behavior can be an advantage in food choice studies where the answers are found in people’s choice behaviors, rather than measuring choice based on intention or “imaginary on paper”. This indeed favors more observational and implicit approaches, which permits consumers to deal with food in a natural way, and this behavioral research can be considered closer to real life.

In order to explain food choices, we as researchers seek to explore various factors that could play important roles in eating behavior. Psychobiological perspectives highlight the relationship between our appetite control system and food choices. Here, homeostatic processes such as hunger and satiety sensations drive eating behavior, reflecting a need or motivation to eat or not to eat [15,16]. Non-homeostatic processes also influence food choices, for instance eating/choosing food for pleasure or because of desires in order to achieve a hedonic reward [17,18,19]. Exploring subjective appetite and diverse subjective sensations and their relationship to choice can provide essential insights into intake behaviors [20,21]. The Merriam-Webster Dictionary defines a “sensation” as a “state of consciousness due to internal bodily changes, e.g., a sensation of hunger” [22]. We therefore argue that subjective sensations relate to one’s interoceptive state. “Interoception” is defined as our perception of internal bodily signals, e.g., hunger, pain, heat, satiety, muscular and visceral sensations [23,24,25,26]. It is furthermore suggested that interoception is the basis for subjective feelings, sensations and self-awareness, representing a subjective evaluation of “how I feel” [18,23]. With reference to this, the study of subjective sensations might contribute important knowledge to the complex area of understanding the diversity in determinants of food choices. 

Research in the area of subjective appetite has moved towards a broader understanding of the concept. It points towards employing a more diverse and holistic approach by which to better understand human eating behavior [26,27,28,29,30]. Specifically, understanding how subjective wellbeing relates to food can contribute to a better understanding of consumer choices [28,31]. Food-related wellbeing and quality of life can be significant additions when conducting consumer food studies [26,27,28,32,33]. Researchers bring in multiple variables when assessing sensations in relation to eating and health [30,34,35,36]. For instance, food studies have used variables such as psychological wellbeing, nausea, physical wellbeing, desire for other foods, sensory specific desires, energy level, sleepiness, relaxation and other subjective evaluations [30,35,37]. However, most of these studies do not relate the sensations to subsequent food choices. Focusing on consumers’ subjective sensations as determinants of food choice, therefore, offers a new basis upon which to predict food choice. However, the importance and implications of subjective sensations relative to food choices have scarcely been researched.

This research study thus seeks to unravel the complex area of determinants of food choice by including subjective sensations as important consumer evaluations for food choice. The overall objective of this study is to quantitatively measure consumers’ subjective sensations, and thereafter, relate these subjective sensations to actual snack choices. Snack choice is assessed with a real, actual choice, hereby applying an implicit behavioral measurement. We believe that this implicit observational measure validates and represents an actual daily snack choice very well. Specifically, the study aims to (1) quantify subjective temporal sensations such as energy, wellbeing, hunger, satiety and desires, and (2) study the effects of subjective sensations on consumers’ choice of specific snacks. Our research questions therefore include:

1. What subjective temporal sensations contribute to snack choice?
2. Do other factors such as gender, time of day and health consciousness affect consumers’ snack choice?

## 2. Materials and Methods 

### 2.1. Participants, Recruitment and Procedure

Participants (*n*_total_ = 253) were recruited from the campus at University of Otago in Dunedin, New Zealand. Participants included undergraduate and postgraduate students ranging from 18−30 years old. Inclusion criteria involved being above 18 years old and in general good health. Exclusion criteria involved having any food allergies. Table 1 displays the characteristics from the 253 participants. Recruitment occurred between 8 am and 4 pm on campus. Students were approached in person and asked if they wanted to participate in a study about appetite sensations. After agreeing to partake, participants were given instructions and handed an iPad to fill in the online questionnaire. After completion of the questionnaire, the iPad was returned to the researcher, and the participants were given the option to choose one snack from a selection of six types snack products. The researchers registered all participants’ snack choices. All participants gave written consent prior to the commencement of the study. University of Otago Human Ethics Committee approved this research (19/018).

### 2.2. Questionnaire 

The choice of sensation variables and the development of the questionnaire were based on existing scientific literature on appetite and sensations, and were also adapted from Duerlund et al. (2019) [26,29,30,36,38].

Sensation measurements were collected using a visual analogue line scale (VAS) via the Compusense^®^ Cloud software (Compusense Inc., Guelph, Ontario, Canada) in randomized order. A continuous scale ranging from 0, i.e., “not at all” to 10, i.e., “very much”, was used to evaluate each sensation. Subsequently, all participants filled out a demographics and psychographics section including gender, age, weight, height, time since last food intake and health consciousness related questions. Health consciousness was evaluated by asking participants the extent to which they agree with the following two statements: “I choose food carefully to ensure good health” and “I think of myself as a health-conscious consumer”, adapted by Squirel et al. (2001) and used also by other researchers evaluating consumer health consciousness [39,40,41]. The responses were collected on a seven point Likert scale ranging from “completely disagree” to completely agree”. Table 2 presents all questionnaire variables including demographic and psychographic background questions.

### 2.3. Snack Samples

A selection of six different snack products which are commercially available in New Zealand was chosen for the study. All snacks were presented unpacked to avoid any brand knowledge related biases. The six snacks were divided into two categories, with each representing a healthy and unhealthy version of a sensory taste profile, sweet, salty or fatty (Table 3). The snacks were chosen based on two criteria: Firstly, from interviews with New Zealand students about their perception of snack products (‘What is a snack?’); and secondly, from screening the available snack products in supermarkets, both through actual physical observation and online research of display and availability. Emphasis was given to choosing snacks with similar levels of familiarity to New Zealand students, and for snacks to reflect a similar price range, so as to avoid any biased choices based on expensive versus cheaper options. To avoid any biases related to portion size, the snacks were presented in portions that were visually comparable and regarded as a proper snack amount. Therefore, the portion size of the snacks depended not on calorie content but on appearance. 

### 2.4. Real Snack Choice

**Figure 1:**
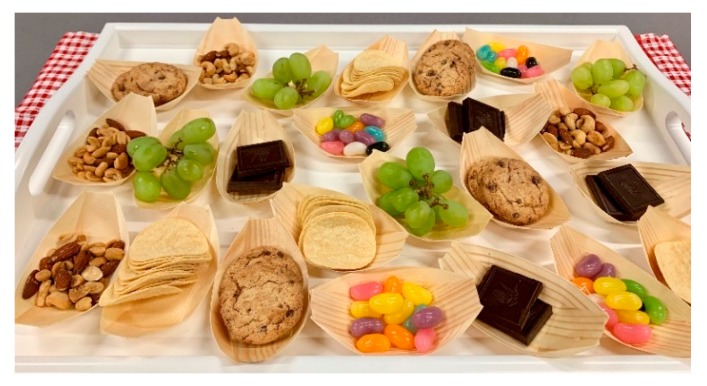
Picture of the six snacks in a randomized display.

Participants were offered a snack of their own choice as a token of thanks for their participation. The researcher noted the real snack choice as an implicit choice measure. The snacks were placed in small containers to avoid the risk of poor hygiene contamination. All snacks were represented four times in a display at all times, so that the snacks were always equally represented to allow choice based on attractiveness rather than availability; see Figure 1. The display was consistently refilled and randomized in an unstructured way to ensure that each participant always had the same amount of choice options. An assistant was appointed to carry out this task with care. 

### 2.5. Data Analysis

Data on self-reported weight and height were used to calculate body mass index (BMI): weight (kg)/(height (m))^2^. One-way analysis of variance (ANOVA) was performed for each sensation (dependent variable) and all snack types (explanatory variable, six levels) to analyze significant snack sample differences for each sensation. *p*-values ≤ 0.05 were considered statistically significant, and a Tukey’s Honest Significant Differences (HSD) test was applied for pairwise comparisons between the six snack types. Effect sizes were examined using Cohen’s d values [42]. A Principal Component Analysis (PCA), including Pearson correlations, was undertaken to reveal the patterns of correlations among the sensation variables. Binomial Logistic regression models were applied separately for each snack choice response in order to examine the effect of the subjective sensation state on the specific choice behavior. Subjective sensation variables functioned as the explanatory variables, and the snack choice as the implicit binary response type (1/0, choice/no choice). Models were iteratively reduced in case of nonsignificant effects to produce more stable models. ANOVA, PCA and logistic regression models were carried out using XLSTAT by Addinsoft, version 2019.2. (XLSTAT, Long Island, NY, USA) [43]. 

Subsequently, the data were organized into three separate data matrices: *X* (sensation measures), *Y* (implicit choice measures) and *Z* (background data) in order to prepare for multivariate L-shaped Partial Least Square Regression (L-PLSR) analysis, as described by Martens et al. (2001) and applied by Giacalone et al. (2013) [44,45]. The X-matrix consisted of the significant subjective sensations as columns and each snack sample as rows. The Y-matrix consisted of the implicit consumer snack choice data (1/0, choice/no choice), with consumers as columns and snack samples as rows. The Z-matrix consisted of the background data, with consumers as columns and background information as rows. Data matrices X (sensations) and Z (background data) shared no dimensions but were connected via a data matrix Y. L-PLSR was employed to find the latent variables modelling the covariance structure between the three data matrices [46,47]. Prior to L-PLSR analysis, preliminary full cross-validated two-block PLSR analyses using Martens’ uncertainty test were carried out to validate the variance and significance of the different sets of variables [48]. The first preliminary PLSR model was performed with X (sensation measures) and Y (implicit choice) containing the actual snack sample choice observations. This two-block PLSR model was performed to reveal the sensations that were significant for explaining snack choice, using a so-called discriminant Partial Least Square Regression (D-PLSR). The second preliminary two-block PLSR model was performed on X (background data) and Y (implicit choice) to reveal the significant explanatory background variables for snack choice. Thus, only specifically chosen and significant variables were included in the L-PLSR analysis. All multivariate PLSR models (incl. L-PLSR) were carried out using Unscrambler^®^ X, version 10.5.1. (CAMO software, Oslo, Norway). 

## 3. Results

### 3.1. Snack Choice Differences

Across the actual snack choices, grapes were chosen most frequently, i.e., by 36% of the participants (*n* = 92). Jelly beans were chosen the least frequently, i.e., by 5% (*n* = 13). Potato chips and dark chocolate were both chosen by 17% of the participants (*n* = 43 and 44), whereas nuts and cookies were chosen by 16% (*n* = 39) and 9% (*n* = 22), respectively. 

One-way ANOVA revealed that consumers’ levels of seven of the thirteen sensations were significantly different for the snack choices: Hunger (*p* = 0.009), Physical wellbeing (*p* = 0.030), Desire-to-eat (*p* = 0.023), Desire-to-snack (*p* = 0.001), Sweet desire (*p* < 0.0001), Fatty desire (*p* < 0.0001), and Salty desire (*p* < 0.0001). Table 4 shows all the details from all sensation variables with least squares means, standard deviations, *p*-values, F-values, and Tukey’s pairwise comparisons. Tukey’s (HSD) pairwise comparison revealed significant differences between the specific snack samples. Specifically, level of Hunger (*p* = 0.029, *d* = 0.5), Desire-to-eat (*p* = 0.011, *d* = 0.6), Desire-to-snack (*p* = 0.003, *d* = 0.7), Fatty desire (*p* < 0.0001, *d* = 1.1), and Salty desire (*p* < 0.0001, *d* = 0.9) were different between consumers choosing grapes compared to potato chips, with medium and large effect sizes [42,49]. Salty desire level was also different between consumers choosing potato chips compared to dark chocolate (*p* < 0.0001, *d* = 0.7), and fatty desire level was different between consumers choosing potato chips compared to nuts (*p* = 0.009, *d* = 0.5). Sweet desire level was different between consumers choosing potato chips compared to cookies (*p* = 0.011, *d* = 1.0), as well as compared to dark chocolate (*p* < 0.0001, *d* = 1.1). Importantly, this ANOVA output does not disclose which sensations were explanatory for the specific snack choice. This will be outlined via the binomial logistic regression analysis explained in Section 3.3.

### 3.2. Patterns of Correlation for Sensations

**Figure 2:**
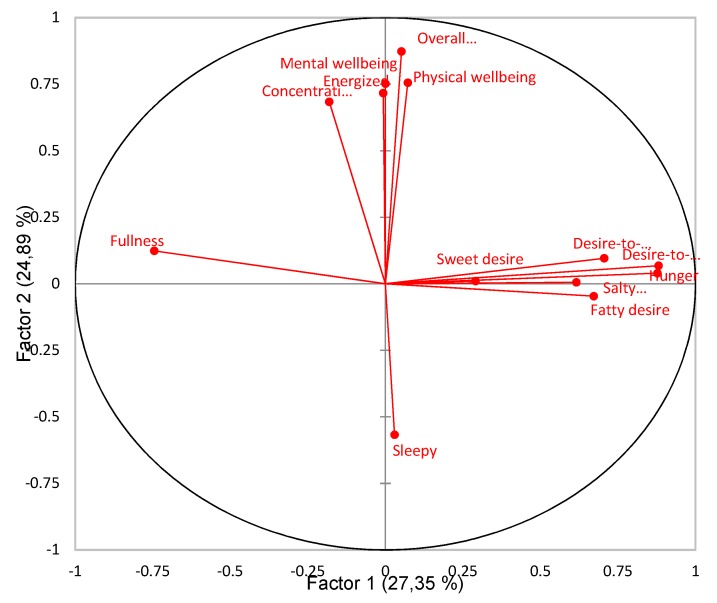
Patterns of correlation for all sensation variables from Principal Component Analysis (PCA) displaying Factor 1 (27.35% explained variance) vs. Factor 2 (24.89% explained variance).

**Figure 3:**
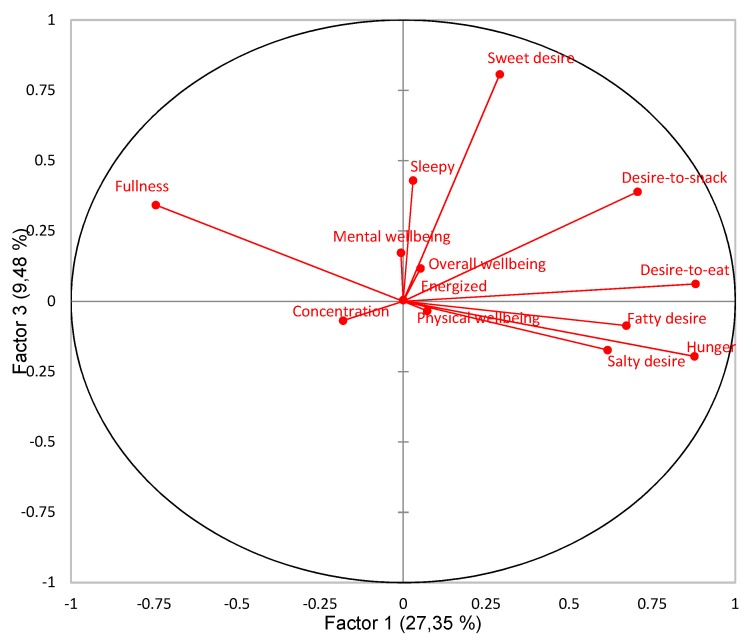
Patterns of correlations for all sensation variables from Principal Component Analysis (PCA) displaying Factor 1 (27.35% explained variance) vs. Factor 3 (9.48% explained variance).

PCA revealed patterns of correlation for consumers’ subjective sensations. A total of 61.72% of the variance was explained in the first three factors. Factors 1, 2 and 3 explained 27.35%, 24.89% and 9.48% of the variance, respectively. The pattern of correlations of Factor 1 vs. Factor 2 (52.24% of explained variance) and Factor 1 vs. Factor 3 (36.83% of explained variance) is presented in Figure 2 and Figure 3, respectively. Results showed four main directions for subjective sensations. Factor 1 clearly separated appetite-related sensations, and Factor 2 clearly separated more vitality- and energy-related sensations, (Figure 2). Factor 3 was included because it revealed a different pattern than the first two factors, namely that Sweet desire mainly explained the variance for the third factor, (Figure 3). Sweet desire was hence not explained well in either Factor 1 or Factor 2, but rather in Factor 3. Sweet desire was positively correlated with Desire-to-snack (*r* = 0.37, *p* < 0.0001), Desire-to-eat (*r* = 0.27, *p* < 0.0001), and Sleepy (*r* = 0.14, *p* = 0.026).

### 3.3. Sensations’ Effect on Choice

**Figure 4:**
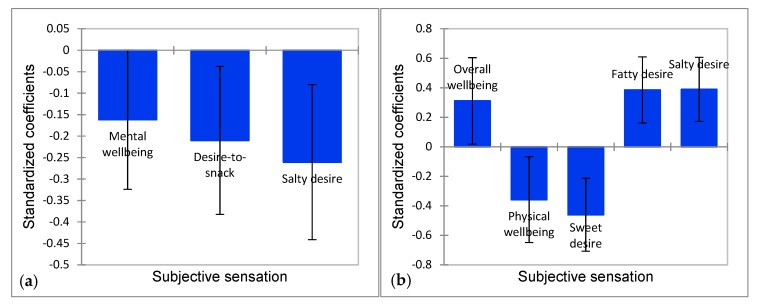
Significant explanatory sensations for (**a**) grapes choice and (**b**) potato chips choice depicting standardized coefficient values including 95% confidence intervals.

In order to examine the effect of subjective sensations on choice behavior, and thereby, to link the specific snack choice to its explanatory sensations, we performed a binomial logistic regression analysis. Figure 4 shows the significant effect of sensations on the two large choice groups: grapes (chosen by 36% of the consumers) and potato chips (chosen by 17% of the consumer), by depicting standardized coefficient values (COEF) including 95% confidence intervals (CI). 

After iteratively reducing the model to increase its stability, we found that three sensations had a significant negative effect for grape choice: Mental wellbeing (*X*^2^ = 3.818, COEF = −0.162, 95% CI [−0.324, 0.00], *p* = 0.050), Desire-to-snack (*X*^2^ = 5.693, COEF = −0.210, 95% CI [−0.382, −0.037], *p* = 0.017), and Salty desire (*X*^2^ = 8.002, COEF = −0.261, 95% CI [−0.441, −0.080], *p* = 0.005). Hence, sensing Mental wellbeing, Desire-to-snack, or a high Salty desire caused consumers *not* to choose grapes. Salty desire had the largest effect of the three sensations. No sensations positively drove grape choice. We found that five of the subjective sensations had an effect on potato chips choice. Sensing Overall wellbeing (*X*^2^ = 4.350, COEF = 0.312, 95% CI [0.019, 0.604], *p* = 0.037), Fatty desire (*X*^2^ = 11.383, COEF = 0.385, 95% CI [0.161, 0.609], *p* = 0.001), or Salty desire (*X*^2^ = 12.394, COEF = 0.389, 95% CI [0.173, 0.606], *p* < 0.0001) showed a positive effect on potato chips choice. On the other hand, sensing Physical wellbeing (*X*^2^ = 5.823, COEF = −0.358, 95% CI [−0.649, −0.067], *p* = 0.016) or Sweet desire (*X*^2^ = 13.288, COEF = −0.460, 95% CI [−0.708, −0.213], *p* < 0.0001) had a negative effect on potato chips choice. Hence, sensing Physical wellbeing or a high Sweet desire caused consumers not to choose potato chips, whereas sensing Overall wellbeing, a high Fatty desire or a high Salty desire caused consumers to choose potato chips. The three desires, i.e., Sweet, Fatty, and Salty, had the largest effects. Table 5 shows the details for all the snack choices as well their significant explanatory sensations with either a positive or a negative effect given by the standardized coefficient value (COEF).

### 3.4. Overall Structure by L-PLSR

**Figure 5:**
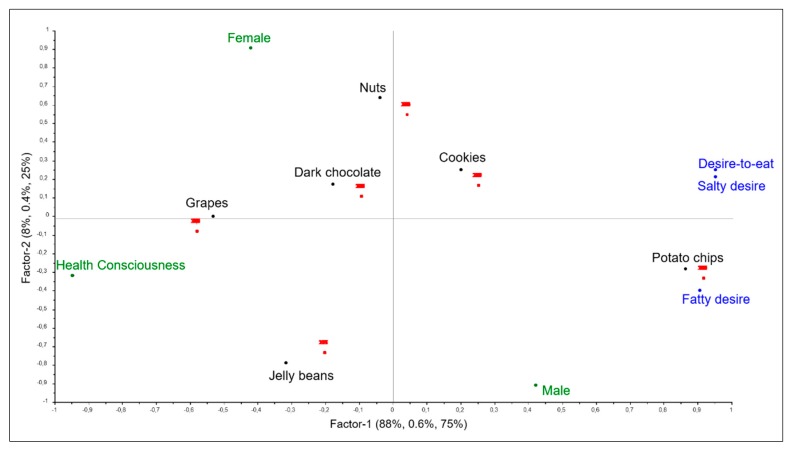
Correlation loadings from L-PLS regression analysis showing consumers’ real choice (**●**) for the six snacks (**●**), explained by both subjective sensations (**●**) and background variables (**●**). The plot displays Factor 1 (X explained variance: 88%, Y: 0.6%, Z: 75%) vs. Factor 2 (X explained variance: 8%, Y: 0.4%, Z: 25%). Variables not explanatory for choice were omitted through preliminary full cross-validated two-block PLS regression analyses.

The overall structure and relationship between subjective sensations, background variables and real snack choice were provided by the L-PLSR analysis. The preliminary full cross-validated two-block PLSR models revealed which variables were retained for the L-PLSR. Consequently, significant sensation variables were Salty desire, Fatty desire, and Desire-to-eat. Significant background variables were Health consciousness, gender female and gender male. The remaining non-significant variables were thus omitted from the L-PLSR analysis. Figure 5 shows the correlation loadings plot from L-PLSR, which visually summarizes the systematic covariation from the multiblock analysis. It illustrates the interrelationships of consumer snack choices (*Y*) explained by both subjective sensations (*X*) and background information (*Z*). Interpreting the plot in Figure 5, we see that consumers’ real choices (**●**), as expected, are represented together with the snack samples given the nature of a choice or no-choice design (1/0 binary response). The consumers are thus grouped together for one snack choice. The results further show that both subjective sensations (**●**) and background information (**●**) bring important knowledge to the overall picture, and thus provide explanations for real snack choices. Factor 1 is explained by Health consciousness, depicted to the left in the plot, as well as desire sensations (Salty, fatty, Desire-to-eat), depicted to the right in the plot. Factor 2 is mainly explained by gender, with Females placed in the upper part of the plot and Males in the lower part. Clustering variables around choice, we observe that the choice of grapes was mainly explained by Health consciousness and gender Female. No sensations positively correlate and explain the choice of grapes. Around the upper left quadrant, we also see a cluster including the three presumed healthy snack options, i.e., grapes, dark chocolate, and nuts. In contrast, we see that the desire sensations and gender Male explain and cluster around the more unhealthy snack options, especially potato chips and partly cookies. Data matrix *X* explained in total 96% of the variance in the L-PLSR analysis, compared to data matrix *Z,* with 100% explained variance, and data matrix *Y* with 1.0% explained variance. This makes sense, considering that both the X- and Z-matrices bring new information to explain the choice and include quantitative variables that show variation amongst consumers, whereas the Y-matrix is a dummy matrix with binary variables that essentially indicates snack choice.

## 4. Discussion

### 4.1. Main Effects of Desires on Snack Choice

In relation to our first research question, i.e., which subjective temporal sensations contribute to snack choice, we found that subjective desires contribute the most, both positively and negatively. The results showed that especially consumers’ sensory specific desires (Salty, Fatty, Sweet desire) discriminated between the snack choices and also had the greatest effects in general. The theory behind Sensory Specific Desires (SSD) can be described as a general desire for certain tastes (e.g., sweet, salty, or fatty foods) and an intrinsic motivation to eat a food that contains that characteristic [19,50]. Both Salty and Fatty desires were explanatory for consumers’ choice of potato chips. Making a choice based on desires might be in line with the previously described impulsive decision-making [4,9,12]. For instance, Honkanen et al. (2012) found that impulsive snacking was characterized by the tendency to buy snacks without thinking [13,14]. This could perhaps reflect a behavior driven by an internal desire. Salty and Fatty desires also showed overall very high positive correlation with each other, as well as with Hunger. It is suggested that desires for specific sensory stimuli may be related to hedonic hunger, and function as a new and different dimension of appetite [26,36,51,52]. Hedonic hunger is driven by the need for pleasure rather than for nutrients, which is the case for homeostatic hunger [51]. We believe that in this research study, consumers’ desires (Salty, Fatty desire, and Desire-to-snack) and, thereby, hedonic hunger, drove choices, especially for the pleasurable hedonic snack choices such as potato chips and cookies, adding importance to non-homeostatic factors for actual snack choice.

Yet, Sweet desire seemed to not be related to the other desires, nor to Hunger nor Fullness, but revealed a different pattern than the other desire sensations, as illustrated in Figure 3**.** Sweet desire, hence, seemed to be disconnected from general appetite-related sensations. Sweet desire was not predictive of any of the presumed sweet snacks either, but instead, had a positive effect on dark chocolate choice and a negative effect on potato chips choice. Harington et al. (2016) suggest that our desire for sweet foods is partially disconnected from our appetite, hypothesizing a “dessert mentality” [53]. This could mean that there is always room for something sweet, regardless of other appetite sensations [26,52]. Harington et al. (2016) found that the desire for sweet was maintained for their whole study period of three hours, after eating two slices of bread, whereas participants’ desire for salty, fatty and savory decreased [53]. This suggests a disconnect of sweet desire from the other desires as well, which is in line with the results in this research study. Thus, including desires when studying food choice and eating behavior in general is highly relevant and provides valuable information. Sensory specific desires were revealed to be important determinants for actual snack choice. However, Sweet desire appeared to have its own agenda and showed different patterns. More detailed knowledge about the disconnect of Sweet desire from appetite and other desires is therefore needed. 

### 4.2. Health Consciousness and Females as Explanatory for Healthy Snack Choice

Multivariate analyses revealed that not only consumers’ subjective sensation state or only consumers’ background information data explained snack choice. Both subjective sensations and background data brought important knowledge to the overall picture. This underlines that we live in a multifaceted world, and that the processes around food choice are complex [3,4]. For this snack study, and in relation to our second research question, Health consciousness was a driving factor for healthy choices, especially grapes. The three presumed healthy snack options in this study, i.e., grapes, nuts and dark chocolate, were mainly explained by gender Female and Health consciousness, whereas male consumers more frequently chose the unhealthy snacks. These results add to plentiful research showing that gender and health consciousness are linked to healthy eating, and that health consciousness itself is a substantial driver of healthy eating in general [5,14,41,54,55,56,57]. Consumers with high health consciousness tend to engage in behavior that improves their health as, well as follow health and dietary recommendations [41,58,59,60]. Additionally, according to Honkanen et al. (2012), reflective or deliberate snacking behavior (as opposed to impulsive) was characterized by an attitude towards unhealthy snacking, and furthermore strengthened by a strong food related self-control [14]. Her and Seo (2017) investigated the role of health-consciousness on the intention to order desserts, finding that highly health-conscious consumers reliably showed low intention [54]. Hartman et al. (2012) examined the associations between snack frequency and healthy versus unhealthy food choices in order to identify underlying patterns. They found that high frequency snacking happened in the context of both healthy and unhealthy lifestyle patterns. However, the unhealthy snacking consumer segments were less health conscious than the healthy snacking segments [61]. Moreover, they found that women chose fruit snacks and made healthier choices than men, while men more often chose unhealthy snacks such as sweets and savories [61]. Amongst adolescent consumers, Mielby et al. (2012), also found that boys chose baked savory and sweet snacks, whereas girls chose fruit snacks such as grapes more often [62]. Consumers in this study were between 18 and 30 years old and revealed similar tendencies. This raises the question of why female consumers choose more healthy snacks in general. Weijzen et al. (2009) found that the aforementioned discrepancy between intention and actual choice was less evident amongst female participants, and that females had a healthy snacking habit as well as strong self-control [5]. Hartman et al. (2012) found that the consumer segment with high frequency healthy snacking habits consisted mainly of females reporting high levels of health consciousness, as well as regular breakfast consumption and low consumption of alcohol in general [61]. Leaning on these findings, we might reason that health-conscious and high self-control females, more often than male consumers, do not follow their immediate desires, but tend to follow a more reflective choice pattern when choosing snacks. The results from this study could also be reflected in this reasoning and support the consistency between health attitudes, intention and actual choice behavior.

### 4.3. Differences in Subjective Wellbeing Sensations

For the included wellbeing sensations, i.e., Overall, Mental and Physical wellbeing, we observed that all three parameters were important and showed significant effects on snack choice, but in different ways. Overall, wellbeing and Physical wellbeing revealed opposite effects for potato chips choice, showing that high Overall wellbeing positively drove that choice, whereas high Physical wellbeing caused consumers not to choose potato chips. Furthermore, consumers that rated high Mental wellbeing showed a negative effect on grape choice. So, even though the three wellbeing sensations showed high positive correlations to each other independently of snack choice (Figure 2), it seems that consumers do distinguish (perhaps subconsciously) between these three sensations before choosing a snack, and that the three wellbeing sensations each bring important knowledge to the overall picture. Food-related wellbeing has gained a lot of interest in recent years [26,27,32,34,36,37,63], but its multifaceted nature proves very difficult to define and conceptualize [28,64,65]. Consequently, there is no one universal way of defining or measuring wellbeing; King et al. (2015) suggest that it includes five dimensions, namely physical, emotional, social, intellectual and spiritual, as encompassed in their developed WellSense Profile™ [34]. Wellbeing in food-related contexts has shown strong associations with physical health when eliciting consumer associations with wellbeing [31,33]. Andersen and Hyldig (2015) also found qualitative evidence that physical wellbeing functions as an important element in food satisfaction [35]. Food-related wellbeing is furthermore recognized as a comprehensive holistic concept that goes beyond just physical health [27,28], and is additionally associated with sensations such as calmness, happiness, satisfaction, joy and general positive emotions [26,33,66]. Psychological wellbeing was also found to be explanatory for food satisfaction in a study by Andersen et al. (2017) [38].

Regular healthy eating results in better wellbeing. Foods that are recognized as positive for wellbeing include fruits, vegetables, grains and dairy products, whereas foods high in fat, salt and sugar are perceived to be negative for one’s wellbeing [33,67]. This applies to the effect of food on wellbeing, but the direct effect of subjective wellbeing for healthy food choices is less clear. Gardner et al. (2014) found that mood influences the choice between healthy and indulgent foods, with positive mood leading to a preference for healthy foods and negative mood leading to a preference for indulgent foods [68]. Somewhat controversially, we found that Overall wellbeing had a positive effect for the presumed unhealthy potato chips snack choice, a snack which is high in salt and fat. Could this mean that if you feel general overall wellbeing, then you choose your snacks based on your desires and not your health attitudes? These findings add to the significance, importance and applicability of evaluating subjective wellbeing in consumer research. Exploring food-related wellbeing can help to clarify some of the factors influencing our food choices, and perhaps try to unravel the complex implications for choice, intake and eating behaviors overall [4,28]. 

### 4.4. Sleepiness Versus Energy

The vitality- and energy-related sensations such as Sleepy, Energized and Concentration did not have any significant effects on the actual snack choice behavior in this study. Thus, in this study and in relation to the first question, these subjective sensations were not important or contributed to actual snack choice. Nonetheless, independently of snack choice measures, we observed that these sensations functioned almost as opposites in consumer perceptions (Figure 2), with Sleepy being significantly negatively correlated to Energized and Concentration. In a previous study done by the authors [36], we observed that sleepiness and energized displayed almost opposite dynamic curves after the consumption of a breakfast meal, with an immediate decrease in sleepiness and an immediate increase in energized, suggesting that feeling energized and sleepy function as two opposites of one pole [36]. Energy level was demonstrated to be an important factor in driving consumers’ food satisfaction in a study by Andersen et al. (2017). They did not measure sleepiness, but found energy to be negatively correlated to nausea [38]. Energy sensations have been found to include both positive sensations such as concentration and feeling alert, but also more negative sensations such as a lack of focus, heaviness and sleepiness [26]. Boelsma et al. (2010) applied and concluded a measure of overall postprandial wellness, which included both pleasantness of sleepiness, physical energy, mental alertness, hunger and fullness. They thus explained these variables in a common underlying factor [30]. With regard to energy and sleepiness, research suggests a term called “post-lunch sleepiness” or a “post-lunch dip” in energy. It occurs, accordingly, after having lunch, and is a distinctive urge for sleep in the early afternoon [26,69,70,71]. According to Monk (2005), this “post-lunch-dip” in energy can occur even without having eaten lunch, or without being aware of the time of day [71]. In the present study, we did not serve any food to the participants prior to the subjective sensation measurements, nor did we allocate the data collection to a specific time of day. Taking the above into consideration, researching consumers’ energy levels in relation to food choices has several applications, including whether eating food influences the sensation of, e.g., sleepiness or energy, or whether these vitality- and energy-related sensations influence the next meal intake or even longer-term food choices. In this study, these sensations did not affect snack choice. Perhaps, if the study had extended data collection to the evening, these sensations would have shown significant effects on snack choice. The applications require more research for elaboration and new knowledge creation.

### 4.5. Research Contribution and Future Perspectives

The present results contribute to the multicomplex area of understanding determinants of food choices and eating behaviors. With this study, we add new knowledge about effects of subjective temporal sensations on actual snack choice. The contribution of the different sensations to snack choice is imperative, and helps to unravel and understand some aspects of snack eating behavior.

While this research study focused on snack choice, future studies should extend the investigation of the effect of subjective sensations on main meals and general food intake. The importance of subjective sensations in relation to predictions of longer-term food intake is, to date, unstudied. Therefore, additional and longer-term research is necessary to support our findings and to gain more in-depth knowledge about the importance of subjective sensations and their relevance to food choice behavior over time. Furthermore, researching subjective sensations with other and more diverse approaches such as including more implicit and indirect measures for both homeostatic and non-homeostatic sensations could add to the validation and application of predicting food choices. This entails interdisciplinary research, involving research areas such as psychology, neuroscience, human nutrition, biology and physiology. This could provide further insights into (over) eating, and have an impact in broader contexts with implications for public health issues and obesity.

### 4.6. Limitations

In the present study, the six snacks were divided into categories each representing a healthy and unhealthy version of a sensory taste profile, being sweet, salty, or fatty. It is important to consider that these categories might be a matter of interpretation, and that consumers might distinguish other sensory profiles than those that we categorized. For instance, we saw that Sweet desire showed an effect on dark chocolate, and that Fatty desire showed an effect on potato chips. Cookies represented an overall higher mean Sweet desire amongst participants than did the Fatty desire. Nuts represented an overall higher mean Salty desire than Fatty desire. These sensations do not necessarily describe the snacks by sensory attributes, but might be an indicator as to how consumers perceive them, given that desires were explanatory for specific snack choices. Furthermore, some consumers consider dark chocolates or nuts as healthy or unhealthy, or perhaps regard potato chips as overlapping in both fatty and salty. Other examples of researchers categorizing choices in consumer food studies include Muñoz-Vilches et al. (2019) [72], who used the concepts of vice and virtue products, characterized into hedonic or utilitarian categories using crisps and green smoothies as choice options. Her and Seo (2017) [54] categorized cake, cookie and ice cream as unhealthy as their excessive caloric value can lead to aversive health consequences. Hence, we see diverse categorizations, and for this reason, one should avoid making firm interpretations based only on category definitions.

Another aspect to consider when interpreting the results is the possibility that the choice outcome could be affected by the consumer’s own answers, e.g., “I answered high salty desire, therefore I should choose potato chips”. Moreover, the consumer group that chose jelly beans was the smallest of all, comprising only 5%. Therefore, any strong conclusions should not be drawn as to the effects for the choice of jelly beans. Furthermore, it is important to consider the nature and composition of the participants. All participants were relatively young adults associated with the university, whose choice behavior may differ from that of the general population. Therefore, more confirmatory studies are necessary with differing population groups in order to generalize the results from this study.

## 5. Conclusions

This research study provided new knowledge about the effects of subjective sensations on actual snack choice. We explored subjective sensations related to appetite sensations, such as hunger and fullness, but also focused on non-homeostatic subjective sensations such as desires, wellbeing and energy sensations. Sweet desire seemed to be a unique sensation not related to appetite nor other desire sensations. The results demonstrated that especially sensory specific desire sensations (Salty, Fatty and Sweet desire) affected, either positively or negatively, snack choice. Furthermore, wellbeing sensations (Overall, Mental, and Physical wellbeing) also showed significant effects on snack choice, each in different ways, with Physical wellbeing contributing positively to the choice of nuts and negatively to the choice of potato chips. The vitality- and energy-related sensations such as Sleepy, Energized and Concentration did not affect snack choice. Additionally, we found that both subjective temporal sensations and background factors brought important knowledge to the overall picture. Gender and Health consciousness drove snack choice as well, with health-conscious females choosing the presumed healthy snacks and males choosing the presumed unhealthy snacks.

Importantly, this study indicates the applicability of subjective sensations in food choice research that focus on diverse determinants. Notably, including desires and wellbeing sensations elucidates important knowledge. The contribution of the different sensations to food choice is thus imperative, and helps to unravel and understand some aspects of snack eating behavior. This could provide further insights into (over)eating, and have implications for public health issues such as unhealthy snacking and obesity. Future perspectives include interdisciplinary research involving implicit approaches to predict choice behavior over time.

## Figures and Tables

**Table 1:** Participant characteristics.

Characteristics	
*n*_total_ Male/female Age (years) Weight (kg) Height (cm) BMI ^1^ (kg/m^2^)	253 98/155 20.5 ± 2.8 (18−30) * 71.0 ± 15.2 (45−125) * 172.0 ± 9.6 (150−195) * 23.9 ± 4.3 (15.9−45.9) *

* Mean ± standard deviation (range); ^1^ BMI = body mass index.

**Table 2:** Questionnaire variables.

Background Information	Sensation Variables	
Gender Age Height Weight Time since last intake Health consciousness	Energy Concentration Sleepiness Fullness Hunger Overall wellbeing	Physical wellbeing Mental wellbeing Desire-to-eat Desire-to-snack Sweet desire Salty desire Fatty desire

**Table 3:** The six snacks used in the study divided into categories.

-	Sweet	Salty	Fatty
**Healthy**	Grapes	Nuts	Dark chocolate
**Unhealthy**	Jelly beans	Potato chips	Cookies

**Table 4:** Least squares means ± standard deviations (*n* = 253) from analysis of variance (ANOVA).

	*p*-Value	F	Cookies	Dark Chocolate	Potato Chips	Nuts	Jelly Beans	Grapes
**Energized**	0.790 (ns)	0.48	4.69 ± 2.2	5.46 ± 2.2	5.11 ± 2.2	5.22 ± 1.9	4.85 ± 1.9	5.25 ± 2.2
**Concentration**	0.748 (ns)	0.54	5.44 ± 2.1	5.71 ± 2.0	5.30 ± 2.2	5.35 ± 1.9	6.05 ± 2.3	5.27 ± 2.2
**Sleepy**	0.674 (ns)	0.63	5.73 ± 2.6	4.90 ± 2.7	4.63 ± 2.6	4.53 ± 2.7	4.89 ± 2.9	4.92 ± 2.9
**Fullness**	0.062 (ns)	2.13	4.69 ± 2.3	4.89 ± 2.6	3.87 ± 2.7	3.99 ± 2.6	6.37 ± 2.9	4.56 ± 2.9
**Hunger**	0.009	3.14	4.66 ± 2.3 ^ab^	4.49 ± 2.6 ^ab^	5.96 ± 2.7 ^b^	5.14 ± 2.9 ^ab^	2.98 ± 2.6 ^a^	4.35 ± 3.2 ^a^
**Overall Wellbeing**	0.265 (ns)	1.30	6.15 ± 2.1	6.44 ± 1.8	6.33 ± 2.1	6.34 ± 1.9	5.27 ± 2.0	5.81 ± 2.1
**Physical wellbeing**	0.030	2.51	5.96 ± 2.0 ^ab^	6.18 ± 2.0 ^ab^	5.65 ± 2.3 ^ab^	6.86 ± 2.0 ^b^	5.22 ± 1.7 ^a^	5.59 ± 2.2 ^ab^
**Mental wellbeing**	0.163 (ns)	1.59	6.42 ± 1.5	6.25 ± 2.0	5.95 ± 2.1	6.26 ± 2.3	5.05 ± 2.4	5.52 ± 2.5
**Desire to eat**	0.023	2.65	6.10 ± 2.5 ^ab^	5.57 ± 2.4 ^ab^	6.58 ± 2.6^b^	5.66 ± 2.8 ^ab^	4.92 ± 3.2 ^ab^	4.80 ± 3.2 ^a^
**Desire to snack**	0.001	4.08	6.56 ± 2.2 ^b^	5.33 ± 2.5 ^ab^	6.31 ± 2.6 ^b^	5.17 ± 2.7 ^ab^	5.58 ± 3.1 ^ab^	4.44 ± 2.9 ^a^
**Sweet desire**	<0.0001	6.03	5.50 ± 2.4 ^bc^	5.72 ± 2.7 ^c^	3.16 ± 2.1 ^a^	3.64 ± 2.3 ^ab^	4.96 ± 3.4 ^abc^	3.94 ± 2.9 ^ab^
**Fatty desire**	<0.0001	4.86	4.41 ± 2.1 ^ab^	3.57 ± 2.7 ^ab^	6.02 ± 2.8 ^b^	4.62 ± 2.6 ^a^	3.07 ± 2.6 ^ab^	3.21 ± 2.5 ^a^
**Salty desire**	<0.0001	8.02	3.89 ± 2.5 ^ab^	3.39 ± 2.3 ^a^	4.98 ± 2.5 ^b^	3.04 ± 2.5 ^ab^	3.67 ± 3.2 ^a^	2.75 ± 2.7 ^a^

Means with different superscript (^a,b,c^) within a row differ significantly (Tukey *p* < 0.05); Degrees of freedom (5, 247); ns = no significant difference; Data was collected on a continuous visual analogue scale (VAS) ranging from 0 to 10.

**Table 5:** The effect of subjective sensations on each snack choice including significant *p*-values and positive/negative coefficient values (COEF) from binomial logistic regression models.

-	Grapes		Jelly Beans		Nuts		Potato Chips		Dark Chocolate		Cookies	
-	Pr > X^2^	COEF	Pr > X^2^	COEF	Pr > X^2^	COEF	Pr > X^2^	COEF	Pr > X^2^	COEF	Pr > X^2^	COEF
**Energized**	-	-	-	-	-	-	-	-	-	-	-	-
**Concentration**	-	-	0.038 *	0.371	-	-	-	-	-	-	-	-
**Sleepy**	-	-	-	-	-	-	-	-	-	-	-	-
**Fullness**	-	-	-	-	-	-	-	-	-	-	-	-
**Hunger**	-	-	0.005 **	-0.604	-	-	-	-	-	-	-	-
**Overall Wellbeing**	-	-	0.012 *	−0.459	-	-	0.037 *	0.312	-	-	-	-
**Physical wellbeing**	-	-	-	-	0.003 **	0.312	0.016 *	−0.358	-	-	-	-
**Mental wellbeing**	0.050 *	−0.162	-	-	-	-	-	-	-	-	-	-
**Desire to eat**	-	-	-	-	-	-	-	-	-	-	-	-
**Desire to snack**	0.017 *	−0.210	0.019 *	−0.440	-	-	-	-	-	-	0.022 *	0.282
**Sweet desire**	-	-	-	-	-	-	0.000 ***	−0.460	0.000 ***	0.347	-	-
**Salty desire**	0.005 **	−0.261	-	-	-	-	0.000 ***	0.389	-	-	-	-
**Fatty desire**	-	-	-	-	-	-	0.001 ***	0.385	-	-	-	-

The ‘−’ denotes non-significant variables removed from the model to produce a more stable model; COEF = standardized coefficient value: negative or positive effect on snack choice; * *p* ≤ 0.05, ** *p* < 0.01, *** *p* < 0.0001; X^2^ = Chi-square.
